# Temporal dynamics and biogeography of sympagic and planktonic photosynthetic microbial eukaryotes during the under-ice Arctic bloom

**DOI:** 10.1093/ismeco/ycaf075

**Published:** 2025-05-07

**Authors:** Clarence Wei Hung Sim, Catherine Gérikas Ribeiro, Florence Le Gall, Ian Probert, Priscilla Gourvil, Connie Lovejoy, Daniel Vaulot, Adriana Lopes dos Santos

**Affiliations:** Asian School of the Environment, Nanyang Technological University, 50 Nanyang Avenue, 639798, Singapore; Centro de Estudos do Mar, Universidade Federal do Paraná, Pontal do Paraná 83255-000, Brazil; Sorbonne Université, CNRS, UMR7144, Team ECOMAP, Station Biologique de Roscoff, Roscoff 29680, France; Sorbonne Université, CNRS, FR2424, Roscoff Culture Collection, Station Biologique de Roscoff, Roscoff 29680, France; Sorbonne Université, CNRS, FR2424, Roscoff Culture Collection, Station Biologique de Roscoff, Roscoff 29680, France; Département de Biologie, Institut de Biologie Intégrative et des Systèmes, Université Laval, Quebec City, QC G1R1V6, Canada; Sorbonne Université, CNRS, UMR7144, Team ECOMAP, Station Biologique de Roscoff, Roscoff 29680, France; Department of Biosciences, University of Oslo, PO Box 1066 Blindern, Oslo 0316, Norway; Asian School of the Environment, Nanyang Technological University, 50 Nanyang Avenue, 639798, Singapore; Department of Biosciences, University of Oslo, PO Box 1066 Blindern, Oslo 0316, Norway

**Keywords:** under-ice bloom, sympagic algae, metabarcoding, 18S rRNA, biogeography

## Abstract

Photosynthetic microbial eukaryotes play a pivotal role as primary producers in the Arctic Ocean, where seasonal blooms within and below the ice are crucial phenomena, contributing significantly to global primary production and biogeochemical cycling. In this study, we investigated the taxonomic composition of sympagic algae and phytoplankton communities during the Arctic under-ice spring bloom using metabarcoding of the 18S rRNA gene. Samples were obtained from three size fractions over a period of nearly three months at an ice camp deployed on landfast ice off the coast of Baffin Island as part of the Green Edge project. We classified the major sympagic and phytoplankton taxa found in this study into biogeographical categories using publicly available metabarcoding data from more than 2800 oceanic and coastal marine samples. This study demonstrated the temporal succession of taxonomic groups during the development of the under-ice bloom, illustrated by an overall transition from polar to polar-temperate taxa, particularly in the smallest size fraction. Overlooked classes such as Pelagophyceae (genera *Plocamiomonas* and *Ankylochrysis*), Bolidophyceae (Parmales environmental clade 2), and Cryptophyceae (*Baffinella frigidus*) might play a greater role than anticipated within the pico-sized communities in and under the ice pack during the pre-bloom period. Finally, we emphasize the importance of microdiversity, taking the example of *B. frigidus*, for which two ecotypes linked to pelagic and sea ice environments have been identified.

## Introduction

Photosynthetic microbial eukaryotes (“microalgae”) are the major primary producers in the Arctic Ocean, dominating both pelagic (phytoplankton) and ice-associated (sympagic) primary production. Sympagic production tends to be lower than phytoplankton production, accounting for 2%–10% of total Arctic primary production [1]. Ice-associated algae undergo a spring bloom that typically occurs a few months before that of the phytoplankton community, when light intensity increases and snow depth is sufficiently thin to allow light transmission through the ice [2]. During the transition from ice cover to open water, when phytoplankton biomass under the ice is low, sympagic communities serve as a rich food source for both ice-associated and early spring under-ice grazers, such as amphipods and calanoid copepods, respectively [3].

The initiation of the under-ice phytoplankton bloom (UIB) typically coincides with the termination of the sympagic algal bloom [4]. The stratification of the under-ice sea surface layer caused by the influx of freshwater from ice and snow melt reduces convective nutrient transport to the ice, inducing nutrient limitation [5]. The decrease of snow cover also leads to changes in the physical environment of the ice, resulting in photo-inhibition and brine flushing of ice algae [2]. In the water, the increase in light resulting from melting snow sets the conditions for the development of the phytoplankton under-ice bloom [5].

The mosaic of Arctic surface marine environments (sea ice, surface melt ponds, open water), each with a range of nutrient and irradiance conditions, harbours complex heterogeneous communities of ice algae and phytoplankton [6, 7]. This includes a variable contribution of different size fractions pico (0.2–3 μm), nano (3–20 μm), micro (> 20 μm) to total biomass and primary production [6]. Sympagic assemblages tend to be dominated by micro-sized (> 20 μm) pennate diatoms of the genera *Nitzschia*, *Fragilariopsis*, *Navicula*, and *Cylindrotheca* [6–8] and by the strand-forming centric diatom *Melosira* and associated epiphytes as a distinct bottom-ice community [9].

Within the phytoplankton community, the Mediophyceae diatoms *Thalassiosira* and *Chaetoceros* are reported as major contributors [10]. Pico-sized Mamiellophyceae, including the genera *Micromonas*, *Bathycoccus*, and *Mantoniella*, are also abundant components of phytoplankton communities both under-ice and in ice-free waters [11–14]. The bloom-forming nano-sized haptophyte *Phaeocystis pouchetii* has also been reported to dominate pelagic communities even under thick snow-covered pack ice [15].

Major changes in the Arctic cryosphere are significantly altering the structure of sympagic and planktonic communities. The most emblematic and well documented impact of climate change in the Arctic is the rapid decline of sea ice cover in terms of area, thickness and age [16–18], reducing the diversity of microbial eukaryotes in sea ice [7]. The northward flow of relatively warm North Atlantic water into the Arctic Ocean has not only amplified the decline of sea ice [19], but also triggered poleward intrusion of phytoplankton species of temperate origin into the European Arctic [16, 20–22]. Warm anomalies in the Atlantic Water inflow to the Barents Sea have been linked to a shift from diatom-dominated phytoplankton communities to dominance by small coccolithophores [23].

Compared to other oceans, the Arctic Ocean is anticipated to experience the high species replacement with invading species displacing locally extinct species [24]. Gains and losses of species in response to the ongoing changes in Arctic habitats (e.g. decrease in sea ice coverage and increased seawater temperature) are likely to induce significant food web reorganization with potential cascade effects [25, 26]. Microbial eukaryotes differ in their thermal tolerance [27], dispersal capacity [28] and ability to exploit new resources [29], which contributes to local abundance and diversity patterns. Therefore, they are natural proxies of community turnover and ecosystem shifts. An extensive Arctic species list can be found in Poulin et al. [30]. Several studies have provided baselines for pan-Arctic communities of dinoflagellates [31], diatoms [32], and mixotrophic flagellates [33]. Two recent studies have linked taxonomically annotated 18S rRNA sequence “metabarcodes” from the Arctic to their biogeographical categorization (i.e. Arctic-temperate, cosmopolitan, etc), thus providing an overview of the biogeography of key Arctic phytoplankton taxa [32, 34].

The present study sought to address two outstanding questions on the ice and under-ice spring bloom phenology. First, we sought to identify the key microalgae taxa driving the community dynamics. To address this question, we analyzed the community structure of three size fractions both in the ice and in the water using 18S rRNA gene metabarcoding. Second, we aimed to assess the biogeographical patterns of ice and under-ice species over the Arctic and beyond by comparing our data with those found in over 2800 publicly available samples. Our results highlight the role of previously overlooked Arctic groups like cryptophytes and bolidophytes and point to a shift from polar to polar-temperate taxa during the development of the under-ice bloom, notably in the smallest size fraction.

## Material and methods

### Study area and sample collection

**Figure 1:**
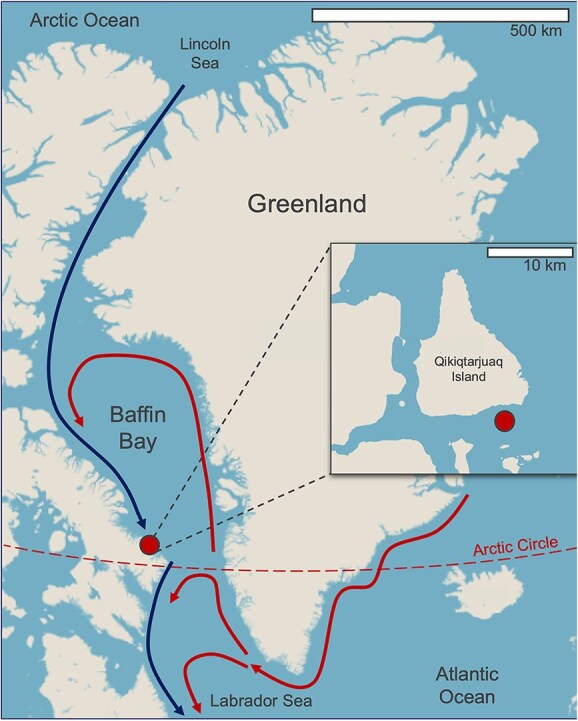
Location of the ice camp on landfast sea ice near Qikiqtarjuaq Island on the western coast of Baffin Bay. Arrows indicate a simplification of the counter-clockwise Atlantic-derived (red) and Arctic-derived (blue) water mass circulation, adapted from Tang et al. (2004).

**Figure 2:**
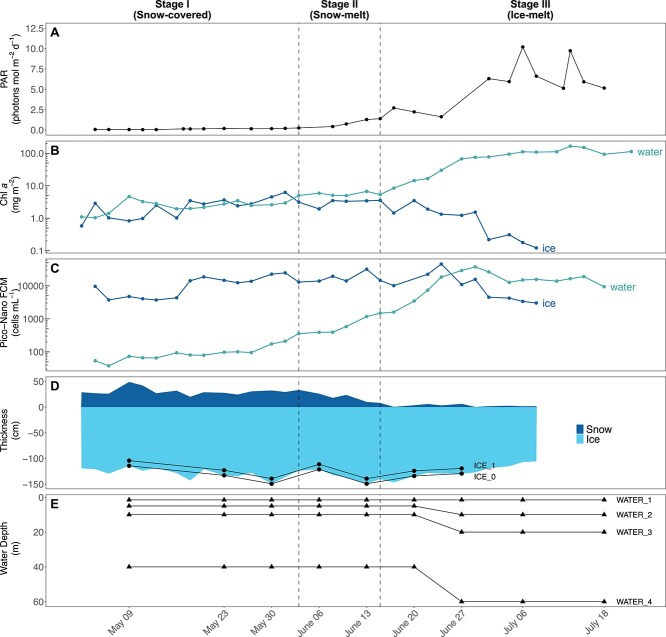
Environmental variables and sampling through the three main stages of the under-ICE phytoplankton bloom. (A) Under-ICE PAR at 2 m depth (Mol photons M^*−* 2^ d^*−*1^); (B) surface integrated Chl *a* concentrations (mg M^*−* 2^) from ICE (bottom 10 cm) and water column (top 60 m); (C) Pico/nano-phytoplankton abundance (cells ml^*−*1^); (D) ICE and snow thickness (cm). ICE_0 and ICE_1 represent samples from the bottom 3 cm and 3–10 cm of ICE; (E) water samples collected at four levels. No data were available for ICE and snow thickness after July 08.

The field campaign was conducted from an ice camp established on the western coast of Baffin Bay southeast of Qikiqtarjuaq Island, Nunavut, Canada (67.4797°N, 63.7895°W, Fig. 1), on landfast sea ice, as part of the Green Edge project. The ice camp was situated away from the shallow shelf, where the water column depth was 360 m. Sampling was carried out every two days between 4 May - 18 July 2016 at an ice camp set up, spanning periods of ice-covered to the point when waters were ice-free. Bottom-ice was collected from two sections of ice cores: (i) the bottom 0–3 cm (ICE_0) and (ii) 3–10 cm from the bottom (ICE_1) of the core (Fig. 2). Under-ice water samples were collected using Niskin bottles at four depths: (i) 1.5 m (WATER_1), (ii) 5–10 m (WATER_2), (iii) 10–20 m (WATER_3), and (iv) 40–60 m (WATER_4) (Fig. 2). Cores were collected using an 8 cm Jiffy corer, and cores placed in sterile bags. Both water samples and cores were returned to the shore based laboratory by snowmobile within a few hours of collection. Ice slices were melted in 0.2 μm filtered seawater. From each sampling depth, 3 L of water and 0.5 L of melted-ice were pre-filtered with a 100 μm mesh and subsequently filtered with a peristaltic pump through the following sets of polycarbonate filters: 20 μm (47 mm), 3 μm (47 mm), and 0.22 μm (Sterivex® filters). Filters were placed in cryotubes, and 1.8 ml of RNALater® was added to either the filters or Sterivex®) units, which were stored at −80°C until processing.

### Environmental and biological data

Variables characterizing the environmental conditions during the time series such as snow and ice thickness, under-water photosynthetically active radiation (PAR). Biological indicators including Chl *a* biomass and photosynthetic pico-nano cell counts were used to group the samples into the three bloom stages described by Ardyna et al. [35]: (I) snow-covered, (II) snow-melt and (III) ice-melt (Table 1, Fig. 2). A brief description of the environmental data used in this paper, and their sampling protocols, are detailed in the Supplementary Information.

**Table 1:** Stages of the Arctic spring bloom in Baffin Bay 2016 ice-camp following Ardyna et al. (2020).

Stage no.	UIB stage	Dates
Stage I	Snow-covered	01 May – 03 June 2016
Stage II	Snow-melt	03 June – 15 June 2016
Stage III	Ice-melt	15 June – 18 July 2016

Species-level identification of diatoms using morphological data was done using scanning electron microscopy (SEM) to complement metabarcoding data in identifying taxa that lack reference sequences. Ice (100 ml) and water (200 ml) samples were filtered through 0.8 μm size polycarbonate (Isopore or Nuclepore) membranes using a vacuum pump (< 250 mm Hg) for scanning electron microscopy. Samples were left to dry in an oven at 35°C for 1 hour and subsequently stored at room temperature.

### DNA extraction, PCR amplification, and sequencing processing

Samples for DNA extraction were selected from each stage of bloom based on the evolution of Chl *a* and photosynthetic cell abundance determined by flow cytometry (Figs. 2B, 2C). The 18S rRNA V4 hypervariable gene region was amplified with the primers TAReuk454FWD1 and V4 18S Next.Rev [36]. PCR purification, library preparation and sequencing was conducted at the GeT-PlaGe platform of GenoToul (INRAE Auzeville, France) using Illumina Miseq (2 × 250 bp). Detailed protocols of nucleic acid extractions and PCR conditions are reported in the Supplementary Information. Sequences were processed with scripts written in the R language [37] using the *dada2* package [38] as described in the Supplementary Information. Amplicon sequence variants (ASVs) were taxonomically assigned using the PR^2^ database version 5.0.1 (https://pr2-database.org, [39]) as a reference. Trophic mode allocation was done using the database of Schneider et al. [40]. All bioinformatic routines including the sequencing processing, trophic mode allocation and culturability are detailed in the Supplementary Information.

### Biogeographical distribution of ASVs

To determine the biogeography of ASVs, we used version 2.0 of the metaPR2 database [41], which contains 18S rRNA metabarcodes from published studies re-processed with *dada2* and annotated with the PR^2^ reference sequence database. ASVs from the V4 region 18S rRNA gene were assigned based on their presence in a total of 2874 marine samples (oceanic and coastal) (Table S1) as polar (≥ 66°N and ≥ 66°S), temperate (23–66°N and 23–66°S) and tropical (23°S–23°N) (Fig. S1).

ASVs from the present study and those from metaPR^2^ were clustered (cASVs) if they showed 100% similarity in their overlap regions (see [41]). cASVs were then assigned to a biogeographical category (e.g. polar, temperate, cosmopolitan), based on their occurrence in metaPR2 samples following the approach of Supraha et al. [32] (Table 2). cASVs were assigned to a biogeography category if at least 90% of the samples where they occurred fell within the regions defined in Table 2. cASVs were assigned as cosmopolitan if they were present in all three major regions (polar, temperate, tropical) but did not have a clear dominance in any region. cASVs that were present in less than five samples were considered unallocated. Multiple samples from a single geographical point (in particular from time series studies) were considered as a single occurrence. In the rest of the paper, we refer to cASV as ASV for simplicity.

**Table 2:** Criteria for biogeography classification of ASVs presented in this study based on their occurrence in 2874 samples from public available datasets gathered in metaPR^2^.

Biogeography	Description	ASV occurrence
Polar	ASV mostly restricted to the Arctic and Antarctic	Polar ≥ 90%
Polar-Temperate	ASV present in the polar and temperate regions	Polar + Temperate ≥ 90%
Temperate	ASV mostly restricted to the temperate region	Temperate ≥ 90%
Temperate-Tropical	ASV present in the temperate and tropical regions	Temperate + Tropical ≥ 90%
Tropical	ASV mostly restricted to the tropical region	Tropical ≥ 90%
Cosmopolitan	ASV has a global distribution	Polar, Temperate, Tropical > 0% each
Unallocated	ASV is either unique to this study or has been found in less than five metaPR^2^ samples	

### Data analysis and visualization

Non-metric multidimensional scaling (NMDS) ordination of a Bray–Curtis dissimilarity matrix was performed with phyloseq [42]. ANOSIM (ANalysis Of SIMilarity) (package vegan, [43]) was used to assess the influence of size fraction, substrate (ice and water) and bloom phase on the community composition. Indicator species analysis (*indicspecies* package, [44]) was performed on ASVs within each size fraction in order to find significant association between taxa and substrate (ice vs. water) and bloom stages (stage I vs. stages II and III). Default *IndVal* index was used as a statistical test with 9999 random permutations. The R packages used for data wrangling and visualization are detailed in Supplementary Information.

## Results

### Environmental parameters

The Green Edge Ice Camp took place in a landfast sea ice on the western side of Baffin Bay south of Qikiqtarjuaq Island (Fig. 1) and spanned periods of ice-covered to ice-free waters. PAR at 2 m from the ice surface (ice-water interface) remained consistently low during snow-covered stage I (Table 1), with values between 0.07–0.25 mol m^−2^ d^−1^ until mid-June (Fig. 2A). Concomitant with the drop in surface albedo of snow, PAR increased from 2.7 to 10.2 mol m^−2^ d^−1^ between stages II and III. Bottom-ice Chl *a* concentrations and sympagic photosynthetic pico-nano (0.2–20 μm) cell concentrations remained stable until the end of the snow-melt period. Bottom-ice Chl *a* concentration peaked at 6.2 mg m^−2^ in early June, while photosynthetic pico-nano (0.2–20 μm) cell concentration reached its maximum value (35 000 cells ml^−1^) by the end of June. The sympagic photosynthetic community then slowly declined towards the end of the sampling period during stage III (Figs. 2B, 2C).

Under-ice Chl *a* concentration and pico-nano phytoplankton cell abundance remained consistently low from May to mid-June (Figs. 2B, 2C). Both parameters peaked (182 mg m^−2^ and 45 000 cells ml^−1^) during the first week of July, when the absence of snow and the presence of melt ponds allowed light to increase (PAR maximum was 10.2 μmol m^−2^, Fig. 2A), setting the conditions for the development of the UIB.

### Community diversity and structure

**Figure 3:**
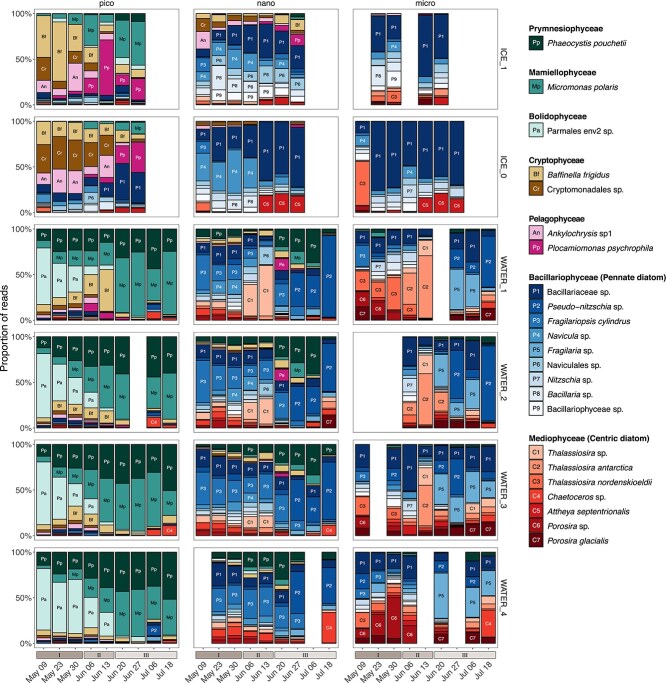
Relative abundance of 18S V4 rRNA reads at the species level from 23 most abundant photosynthetic taxa across bloom stages I to III in ice and water. These species represent 75% of all reads. Samples are sorted according to their size fractions: Micro (20–100 *μ*m), nano (3–20 *μ*m) and pico (0.2–3 *μ*m). Taxa with a minimum of 10% relative abundance of samples are annotated with a two character label. No ice samples were collected in July when melt ponds were formed and the ice was unstable. Other blank columns represent samples lost during processing.

Sympagic and under-ice planktonic communities of microbial eukaryotes were separated by size fractions (0.2–3 μm, 3–20 μm and > 20 μm) and their diversity was assessed by metabarcoding of the V4 region of 18S rRNA gene. In total, 428 ASVs were assigned to photosynthetic taxa. The ice algal and phytoplankton communities were composed of 18 and 21 classes, respectively. They included Bacillariophyceae, Mediophyceae, Pelagophyceae, Chrysophyceae and Bolidophyceae from the division Stramenopiles, Mamiellophyceae, Pyramimonadophyceae and Chlorophyceae from Chlorophyta, Prymnesiophyceae from Haptophyta and Cryptophyceae from Cryptophyta (Fig. S2). Morphological identification using Scanning Electron Microscopy of Bacillariophyceae and Mediophyceae taxa from both sympagic (Fig. S3) and phytoplanktonic communities (Figs. S4 and S5) was used to complement the metabarcoding data. Few genera and species were identified by both methods (Tables S2, S3). Twenty species were only identified by SEM, but not by metabarcoding, for ice (9) and water (11). The majority of ASVs in this study had no match to public sequences from verified cultures (Fig. S6). Community analysis at the ASV level using NMDS and ANOSIM showed that samples clustered according to size fractions along the first axis and substrate (ice and water) along the second axis (Table S4, Fig. S7). Community composition was significantly different among the three stages of the UIB for both ice (R = 0.15; *P* = .004) and water (R = 0.420, *P* = .001) (Table S4). Twenty-three key taxa accounting for 75% of the total photosynthetic reads were selected for analyses of community change across bloom stages and size fractions (Fig. 3).

During the snow and ice covered stages (I and II), the sympagic pico-sized community was dominated by the Cryptophyceae *Baffinella frigidus* and an undescribed Cryptomonadales clade in the 3–10 cm ice layer (ICE_1) and bottom 3 cm (ICE_0), respectively. At the same time, in (ICE_0), *Ankylochrysis* (Pelagophyceae) and *Plocamiomonas* (Pelagophyceae) co-dominated in the bottom ice layer. During stage III, the Mamiellophyceae (*Micromonas polaris*) was abundant in the 3–10 cm ice layer (ICE_1) (Fig. 3). Among pico-sized phytoplankton, the dominant taxon during stage I belonged to Parmales environmental clade 2 with a contribution increasing with water depth (Fig. 3). From stage II onwards, it was replaced by *M. polaris* and the haptophyte *P. pouchetii*. In the surface layer (WATER_1), the cryptophyte *B. frigidus*, which was present in the ice earlier, was abundant during stage II.

The nano-sized communities, both in the water and ice, were dominated in terms of relative abundance and diversity by Bacillariophyceae (pennate diatoms). In the ice, during stage I, a diverse community of nano-sized pennate diatoms inhabited the 3–10 cm layer (ICE_1) without clear dominance of any genus or clade (Fig. 3). Within the bottom layer (ICE_0), *Navicula* and unassigned Bacillariaceae ASVs co-dominated the community (Fig. 3) until the beginning of stage II. From this point, several ASVs assigned to undescribed pennate diatoms (Bacillariaceae) co-dominated the nano-sized sympagic community until the end of the sampling period. There was also a small contribution to the sympagic community from the centric Mediophyceae species *Attheya septentrionalis* during the transition from stage II to III (Fig. 3). A pennate-centric-pennate diatom temporal succession was observed for the nanoplankton in the surface layer, featuring the genera *Fragilariopsis* (*F. cylindrus*), *Thalassiosira* and *Pseudo-nitzschia*. The haptophyte *P. pouchetii* also contributed significantly to the nano-sized community in the water column during stage III.

The micro-sized ice community from stages I and II was also dominated by undescribed Bacillariaceae diatoms, except for bottom ice at the start of stage I when *Thalassiosira nordenskioeldii* was the dominant taxon (Fig. 3). Microplanktonic diatoms were co-dominated by genera and undescribed Bacillariophyceae and Mediophyceae (Fig. 3). The diatom genus *Thalassiosira* (*T. antarctica* and *T. nordenskioeldii*) increased its contribution in the surface layer (WATER_1) during stage II, while *Porosira* had a high contribution to the deep water layer community during stage I (Fig. 3). Among pennate diatoms, *Pseudo-nitzschia*, *Fragilaria* and undescribed Bacillariaceae dominated the microplanktonic community during stage II without a clear pattern in abundance (Fig. 3).

### Biogeography and microdiversity

**Figure 4:**
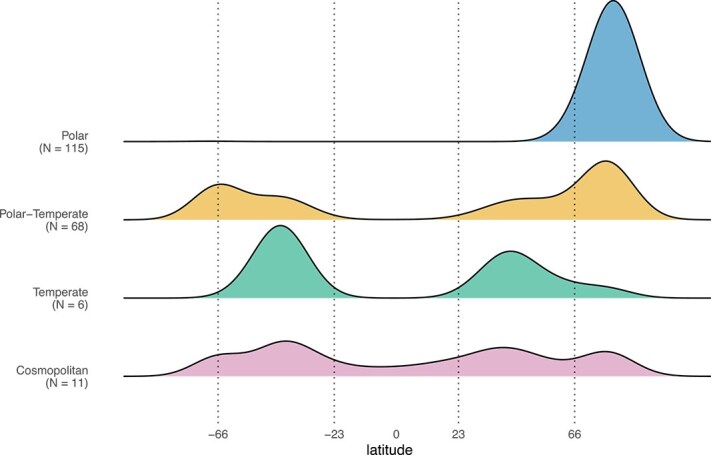
Latitudinal distribution of the metaPR^2^ samples where Green Edge ASVs were observed. Polar, polar-temperate, temperate, and cosmopolitan refer to the biogeographic classification of the ASVs (Table 2. *N* indicates the number of assigned ASVs in each category. The major latitudinal boundaries are illustrated by dashed lines: Arctic circle (66*^°^*N), tropic of cancer (23*^°^*N), tropic of Capricorn (23*^°^*S) and Antarctic circle (66*^°^*S).

We explored the occurrence of the ASVs found in this study across 2874 marine samples selected from the metaPR^2^ database (Fig. S1, Table S1) and classified them according to their latitudinal occurrence (Table 2). A total of 200 ASVs, representing 82.5% of the photosynthetic reads, could be assigned to a biogeographical region (Fig. 4). Most of the assigned ASVs had polar and polar-temperate distributions, with few temperate and cosmopolitan ASVs (Fig. 4). The sympagic communities were dominated by polar ASVs in all size fractions, and also harboured most of the unassigned ASVs. The pico-phytoplankton community was initially co-dominated by polar and polar-temperate ASVs (Fig. S8), but as the bloom developed, polar-temperate ASVs represented most of the reads. In contrast, the nano fraction was dominated by polar-temperate ASVs with no clear change over time. Finally, among the micro-sized phytoplankton, polar ASVs were important at almost all times, except during the last sampling week (stage III) in surface waters (WATER_1 and 2), where polar-temperate ASVs became dominant (Fig. S8).

**Figure 5:**
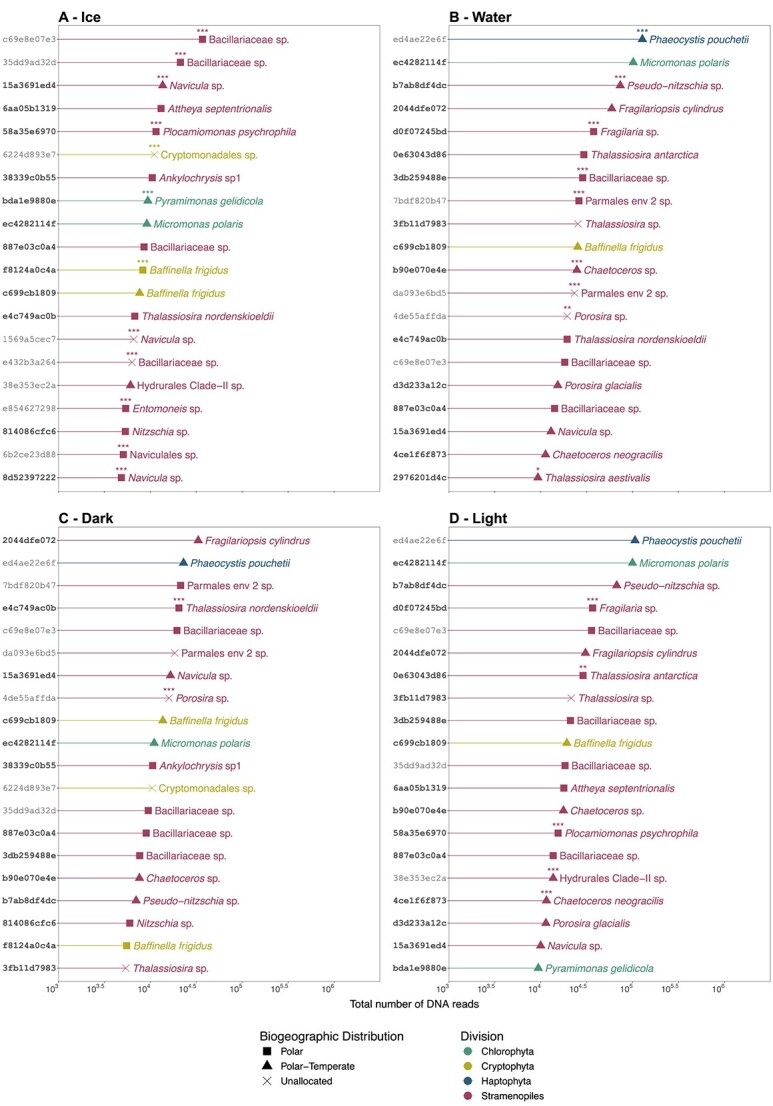
Twenty most abundant photosynthetic ASVs in ice (A) and water (B) substrates, and UIB dark (stage I, C) and light (stages II + III, D) phases. Symbol shape corresponds to biogeographical classification. ASVs in bold correspond to those with sequence matching 100% to PR^2^sequences obtained from cultures. Colours correspond to division. The *indicspecies* statistical significance is shown as follows: ^*^P < 0.05, ^*^^*^P < 0.01, ^*^^*^^*^P < 0.001.

**Figure 6:**
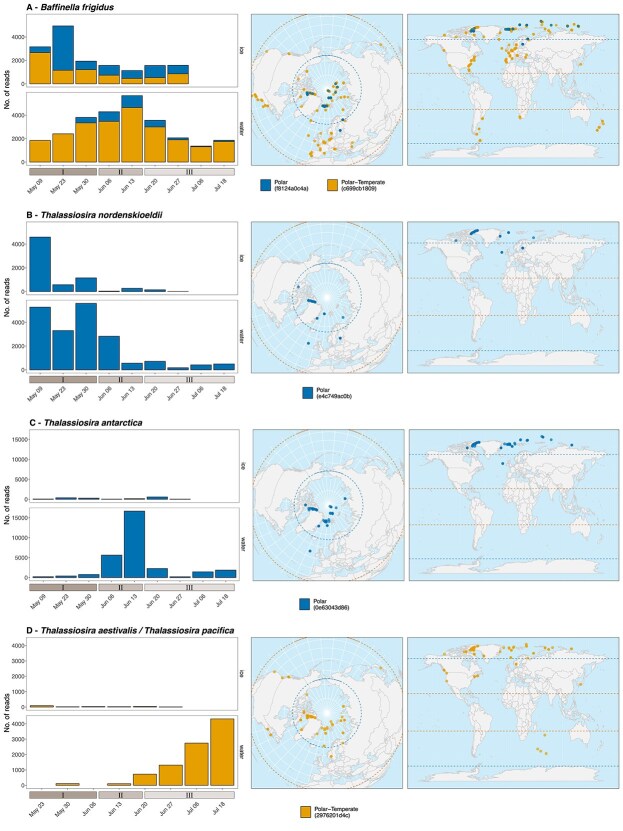
Temporal evolution and geographical occurrence of ASVs assigned to (A) *B. Frigidus*, (B) *T. Nordenskioeldii*, (C) *T. Antarctica*, and (D) *T. Aestivalis/T. Pacifica*. Left: ASVs read numbers across different bloom stages in the ice and water samples (all depths and size fractions combined). Center and right: metaPR^2^ samples where the ASVs were detected. Dashed lines correspond to the polar circles and the tropics.

We then used indicator species analysis to investigate whether the 20 most abundant ASVs were associated with a specific substrate (ice or water) or bloom stage. To facilitate pairwise comparison, the samples from bloom stage I were considered as “dark phase” and those from stages II and III as “light phase” (Fig. 5), based on PAR levels (Fig. 2A). Twelve and nine ASVs were significantly associated with ice and water, respectively. Over half of the ice associated ASVs (7) were assigned as polar (Fig. 5A), while those flagged as indicators of the water community were equally split as polar, polar-temperate and unassigned (Fig. 5B). Fifty percent of the ASVs flagged as indicators of ice or water substrates had 100% matches to at least one culture sequence from an algal culture (Figs. 5A, 5B). Only 2 ASVs were flagged as indicators for the dark phase (the diatoms *T. nordenskioeldii* ASV_e4c749ac0b and *Porosira* sp. ASV_4de55affda) (Fig. 5C) and 5 ASVs were significantly associated with the light phase (Fig. 5D). Among these, 4 had a 100% match to at least one cultured algal sequence (Figs. 5C, 5D). ASVs flagged as indicators from the genera *Baffinella* and *Thalassiosira* that exhibit diversity at the species or ASV level (Fig. 5) were further investigated (Fig. 6A).

The cryptophyte *B. frigidus* ASV_c699cb1809 (polar-temperate) and ASV_f8124a0c4a (polar) are differentiated by a single base pair (Fig. S9). Both were co-dominant in the ice samples, whilst the former was dominant in the water samples (Figure6A). The polar ASV_f8124a0c4a was flagged as an ice indicator (*P* < .001, Fig. 5A). While the polar-temperate ASV_c699cb1809 was more abundant in water samples (Fig. 6A), this ASV was not significantly associated with either ice or water (Fig. 5). The contribution of the ice and polar ASV_f8124a0c4a increased in the water column during the transition between stages II and III (Fig. 6A), possibly due to the release of cells from the ice during melting.

Three *Thalassiosira* ASVs, flagged as indicators and with distinct biogeographical allocation, exhibited a succession pattern during the UIB (Figs. 6B-D). *T. nordenskioeldii* ASV_e4c749ac0b and *T. antarctica* ASV_0e63043d86, both categorized as polar, were flagged as dark and light bloom phase indicators, respectively (Figs. 5C, 5D), while the polar-temperate *T. aestivalis*/*T. pacifica* ASV_2976201d4c (*T. aestivalis* and *T. pacifica* share an identical V4 sequence, so both species names were used to refer to this ASV) was associated with water (Figs. 5B). Based on read numbers, *T. nordenskioeldii* ASV_e4c749ac0b was more abundant in the water during the dark phase and then was replaced by *T. antarctica* ASV_0e63043d86 at the start of the light phase (Figs. 6C, 6D). Finally, the polar-temperate *T. aestivalis*/*T.pacifica* ASV_2976201d4c became the dominant *Thalassiosira* ASV in the water as the under-ice bloom developed (Fig. 6D).

## Discussion

### Taxa succession and diversity

The Green Edge ice camp time series documented the taxonomic succession of sympagic and under-ice phytoplankton communities in southwestern Baffin Bay. During the snow-covered and snow-melt stages, photosynthetic biomass, as indicated by Chl *a* and chlorophyll fluorescing pico-nano (0.2–20 μm) cell concentrations, was detected both in and beneath the landfast sea ice but did not increase as light did not increase during this period (Fig. 2). Snow melt allowed more light to reach the water below the ice, which was marked by an increase in biomass and number of pico and nano phytoplankton cells (Fig. 2). Similar to other time series studies under landfast ice [4], light availability and upper water column stabilization were the key factors associated with triggering the under-ice bloom during the Green Edge campaign [5]. A similar trend was also reported during a simultaneously occurring campaign in the marginal ice zone of Baffin Bay [45, 46].

Community analysis at the ASV level demonstrated that the sympagic and under-ice planktonic communities were significantly different between the three stages of the under-ice bloom (Table S4). Sympagic and planktonic pennate diatoms are important members of under-ice blooms and are able to use low-light levels [47]. In the present study, pennate diatoms, in particular raphid pennate genera such as *Navicula*, *Nitzschia*, *Pseudo-nitzschia*, and ribbon forming genera such as *Fragilariopsis* and *Fragilaria*, were more diverse than the other photosynthetic groups and dominated both sympagic and planktonic nano/micro-sized communities in terms of relative abundance through time (Fig. 3). These taxa are commonly reported in bottom-ice samples [7, 30, 48]. In contrast, the increased contribution of the epiphytic centric genus *Attheya* during snow melt (Fig. 2, Fig. 3) was consistent with studies reporting the appearance of *A. septentrionalis* in bottom-ice communities as light intensities increase through spring [49–51].

Arctic diatom assemblages are often described by their seasonal succession pattern from pennate to centric species, linked to different light requirements [6, 52]. Among nanoplanktonic diatoms, a pennate-centric-pennate succession was observed in the surface layer, where the relative abundance of the centric diatoms *Thalassiosira* increased during stage II and receded during stage III (Fig. 3). The succession observed at the ice camp was, however, not observed in under-ice samples collected during the Green Edge oceanographic campaign [46], which occurred simultaneously offshore in central Baffin Bay [53]. The under-ice community during the Green Edge oceanographic campaign was co-dominated by centric and pennate diatoms, while centric diatoms were associated with the development of the bloom in the marginal ice zone and open waters [46, 54]. These varying temporal dynamics within Baffin Bay highlight the differences in bloom phenology consistent with the heterogeneity of Arctic outflow shelves [55].

Compared to diatoms, pico-sized sympagic and under-ice photosynthetic eukaryotes (≤ 3 μm) have received less attention [13, 56, 57]. Cryptophytes, represented by the genus *Baffinella* and an undescribed Cryptomonadales clade, were among the dominant taxa within the pico-sized sympagic community during the snow-covered stage (Fig. 3). Cryptophytes are able to adapt to a range of light intensities [58, 59]. A time series from the high Arctic Kangerluarsunnguaq fjord (Kobbefjord) on the west coast of Greenland, also found cryptophytes and other flagellates dominating the sympagic community when light was limited [60]. Also during pre-bloom conditions (stage I), Bolidophyceae (Parmales environmental clade 2), a group reported to be abundant in polar and subarctic regions from DNA sequences and microscope observations [61–63], were the dominant picoplanktonic taxa at all depths (Fig. 3). The dominance of cryptophytes and bolidophytes within the sympagic and under-ice planktonic communities under low light regimes indicates their likely role as primary producers during periods of heavy snow cover (winter and early spring). Cryptophytes have been reported as a rich food source for both ice and early season grazers [3, 64–66]. In contrast, the contribution of Bolidophyceae to the polar food web has not been established, although cells resembling the silicified Bolidophyceae have been reported in fecal pellets of subarctic copepods [67, 68].

During the snow-melt stage, a shift in dominance from Bolidophyceae to Mamiellophyceae, represented by the genus *Micromonas*, was observed within the planktonic pico-sized community, except in the surface water layer. The increase in subsurface PAR (Fig. 2) combined with a slight increase in water temperatures and in stratification [5], likely favoured the growth of *Micromonas*. This is supported by the fact that *M. polaris* CCMP2099 has a higher maximum growth rate (0.55 division. Day^−1^) than the cryptophyte *B. frigidus* CCMP2045 (0.40 division Day^−1^) when light is not a limiting factor [13]. Shorter-term physiological experiments have also shown that the optimal growth rates for *Micromonas* were also achieved at a slightly higher temperature range (6°C - 8°C), than *Baffinella* (4°C - 6°C) [13, 69].

Along with *M. polaris*, *P. pouchetii* also dominated the under-ice bloom pico-size community from the snow-melt stage to the end of the time series (Fig. 3). This species is recognized as an important member of the pan-Arctic under-ice community during the spring-to-summer transition [6, 15, 70]. It has also been reported in early blooms beneath snow-covered pack ice [71]. In our data, a small increase in the abundance of *Phaeocystis* in the nano-size community was observed during the early weeks of the ice-melt stage (Fig. 3), which could be due to the formation of cell aggregates [72] or of colonies in the late stages of the spring bloom. Under high-light and low-nutrient conditions, such as those found during the ice-melt stage [5], *Phaeocystis* can form polysaccharide-based mucilaginous colonies that can be millimetres in diameter, with mucilage presumable involved in energy storage and defence against grazers [70, 73]. The *P. pouchetii* ASV obtained during the Green Edge oceanographic campaign was flagged as an indicator of the marginal ice zone and open water sectors within the > 20 μm size fraction, corroborating our results [46].

The considerable amount of novel molecular diversity found in this study emphasizes the need for additional research to genetically characterize arctic taxa. This is illustrated by the fact that most (14) of the diatom taxa observed by SEM lack sequence information and therefore cannot be identified by metabarcoding (Tables S2, S3, Figs. S3, S4 and S5). This parallels the observation that among the 1000 genera catalogued by Fourtanier and Kociolek [74] and Fourtanier and Kociolek [75], only 197 have reference 18S rRNA sequence in the PR^2^ database [39]. Moreover 50% of the most abundant ASVs in our study do not have a cultured representative (Fig. 5), indicating that although culturing methods have been able to capture some of the diversity among arctic taxa [32, 76–78], difficulties remain for isolating and maintaining diatoms in culture (Fig. S6), highlighting the need for renewed culturing efforts and development of novel isolation methods.

### Biogeography and niche preference

The poleward flow of warmer Atlantic and Pacific waters will potentially induce an ecosystem shift in the Arctic Ocean towards a more temperate state, marked by the intrusion of temperate species [22, 79, 80]. Previous studies have linked taxonomically annotated 18S rRNA sequence “amplicons or barcodes” found in the Arctic with their global occurrence in an effort to detect ongoing shifts within the (phyto)plankton community [32, 34]. In the present study, the prevailing geographic distributions of most of the ASVs for which it could be assigned (representing 82.5% of the reads in our dataset) were polar (57.5%) and polar-temperate (34%). This contrasts with a previous study by Ibarbalz et al. [34] which reported, using different approaches (Swarm OTUs vs. ASVs), that although abundant, only 12% of their OTUs were represented by taxa with polar distribution. The high prevalence of polar ASVs in our study may be attributed to several factors. Firstly, the sample set of Ibarbalz et al. [34] only covers the Tara Ocean expeditions (a couple of hundred samples) which is much less extensive than ours (> 2800 samples), limiting the validity of biogeographical inferences. Secondly, their study included Arctic samples collected in the Atlantic and Pacific inflow shelves, where a high number of non-polar barcodes were detected. In contrast, our sampling site was located on the western coast of Baffin Bay, a region characterized by the outflow of modified Pacific and Arctic waters [6]. The under-ice water column at the ice camp was dominated by Arctic Water advected southward along Baffin Island [5]. Finally, in contrast to the study of Ibarbalz et al. [34], the presence of sympagic communities and taxa present at the transition between the late winter and early spring in our dataset might have contributed to an increased number of polar barcodes.

In our study, most sympagic ASVs were classified as polar. Some exceptions (39 of 306) corresponded to ASVs found at latitudes below the polar circle from the Baltic Sea and Hudson Bay, which are seasonally ice-covered [81]. The sympagic community also exhibited more ASVs significantly associated with ice as a substrate than water (Fig. 5). These ASVs may represent sea ice specialists which can cope with the strong gradients of salinity, temperature and light found in the ice [82]. For example, some cryptophyte species like *B. frigidus* have been characterized as euryhaline, growing at salinities ranging from 5 to 35 [69]. The *B. frigidus* ASV_c699cb1809 and ASV_f8124a0c4a were 100% similar to the sequences obtained from the strains CCMP2045 (GQ375264) and RCC5289 (OR736128), isolated from Baffin Bay waters [69] and sea ice, respectively [78]. The latter ASV (ASV_f8124a0c4a) was flagged as an indicator of ice (Fig. 5A) and showed a restricted polar distribution (Fig. 6) which suggests that it probably represents an ice ecotype. Additional studies addressing the intraspecific variability in growth optima between ice and water isolates are required for an in-depth characterization of the niche preferences and fitness of the cryptophyte genus *Baffinella*.

Another example of potential ice specialist taxon is represented by the polar ASV_58a35e6970 also flagged as an indicator of the ice community (Fig. 5A). This ASV is 100% similar to the sequence of the recently described Pelagomonadales species *Plocamiomonas psychrophila*, isolated from sea ice [83]. Metatranscriptomic analysis has revealed that strain CCMP2097 of *P. psychrophila* possesses specific adaptations to cold saline conditions, such as those found in sea ice micro-environments. This includes differential expression of several antifreeze proteins, an ice-binding protein, and an acyl-esterase involved in cold adaptation [84] and the capacity to rapidly adjust to low salinity associated with ice melt [85].

As the bloom developed, we observed a transition from polar to polar-temperate ASVs in the smallest size fraction, while polar ASVs represented by undescribed groups of pennate (Bacillariophyceae) and centric (Mediophyceae) diatoms were major contributors to the micro-sized planktonic community across all bloom stages and nearly all depths (Fig. 3). Body size plays an important role in determining spatial patterns for planktonic organisms, although exact mechanisms are still unclear [86]. For example, some small-sized cells such as the prasinophyte *Bathycoccus* have an extremely broad distribution from the subtropics to the pole [12, 87]. In contrast, Richter et al. [88] showed based on metagenomic data that smaller size classes had more local distributions than larger ones. In our data set, the predominance of polar ASVs within the micro-sized planktonic communities might be explained by the lower dispersal rate of larger cells compared to the pico- and nano-sized taxa. Diatoms associated with sea ice in the Arctic have been reported as endemic taxa [30, 89], and our results corroborate the idea that Arctic endemism is also found for planktonic diatoms [90], especially within the micro-plankton where less connection between distant diatoms communities is expected.

A few ASVs, in low abundance, were assigned as temperate (6) and cosmopolitan (11), some representing taxa with broad biogeography signatures (Fig. 4). The cosmopolitan *Phaeocystis* ASV_8050f737e4 was 100% similar to sequences obtained from the species *P. jahnii* which was initially described from the Mediterranean Sea [91] and reported in the warmer waters of the southeastern East China Sea [92]. The ecological versatility (i.e. broad geographic distribution with species found across several gradients of temperatures and nutrient conditions) of this genus stemming from their ability to grow mixotrophically [93, 94] aligns well with suggestions of a shift towards *Phaeocystis*-dominated blooms in future Arctic scenarios, which will have implications for phytoplankton community structure and trophic energy transfer [15, 95].

While barcodes found in the Arctic but occurring also elsewhere in the metaPR^2^ global dataset may represent indicators of species displacement within the Arctic planktonic community, they also stress the limitation of using barcode sequences, such as the variable regions of 18S rRNA gene, when describing biogeographic patterns of plankton species. The *B. prasinos* ASV_4580ad6202 was assigned as cosmopolitan and although the genus *Bathycoccus* is considered cosmopolitan, the analysis of metagenomes [96], of nuclear genomes [87, 97] and of the internal transcribed spacer 2 [98] have suggested the existence of distinct *Bathycoccus* ecotypes and species, including a polar genotype [97], all of which have identical 18S rRNA sequences and therefore cannot be discriminated by metabarcodes from the V4 or V9 regions of the 18S rRNA gene. Among diatoms, the polar-temperate ASV_2976201d4c represents at least two distinct *Thalassiosira* species, *T. pacifica* and *T. aestivalis*, which share identical 18S rRNA V4 regions and can be only distinguished by scanning electron microscopy. Indeed, *T. pacifica* was detected by SEM in our samples while *T. aestivalis* was absent. The use of more resolutive taxonomic markers, such as ITS or full rRNA gene, capable of distinguishing cryptic genotypes combined with microscopy approaches such as SEM will be required for deciphering complex biogeography patterns [90].

Finally, a large proportion of ASVs (53%), especially originating from the sympagic community, remained unassigned, probably due to the lack of sufficient metabarcoding datasets from ice environments (Fig. S8) in the metaPR^2^ database. Some of our barcodes had a strong occurrence in the Arctic (Fig. 4). However, due to the limited number of datasets available from the Antarctic, we opted for a conservative approach by not separating the polar biogeography categories into Arctic and Antarctic categories. Some of our barcodes may indeed represent bipolar taxa, [99–101] while others maybe truly restricted to the Arctic. Only more extensive sampling from Antarctic regions and multigene phylogenies will be able to solve this question.

## Supplementary Material

Sim_et_al_GE_IC_Supplementary_accepted_ycaf075

## Data Availability

Raw Illumina sequences were deposited to GenBank under project PRJNA810431. All codes and data used in this study can be found in https://github.com/clarencesimple/SIM_GreenEdge_IceCamp/.
