# Supplementary material for: Temporal dynamics and biogeography of sympagic and planktonic photosynthetic microbial eukaryotes during the under-ice Arctic bloom
Source: ISME Commun. 2025 May 7;5(1):ycaf075. doi: 10.1093/ismeco/ycaf075 (PMC12121357; doi:10.1093/ismeco/ycaf075)
Supplement: Sim_et_al_GE_IC_Supplementary_accepted_ycaf075 [file sim_et_al_ge_ic_supplementary_accepted_ycaf075.pdf]

Temporal dynamics and biogeography of sympagic and planktonic photosynthetic microbial eukaryotes during the under-ice Arctic bloom

Supplementary Information

Clarence Wei Hung Sim[1,*], Catherine Gérikas Ribeiro[2], Florence Le Gall[3], Ian Probert[4], Priscilla Gourvil[4], Connie Lovejoy[5], Daniel Vaulot[3,6], Adriana Lopes dos Santos[1,6,*]

[1] Asian School of the Environment, Nanyang Technological University, 50 Nanyang Avenue, 639798, Singapore.

[2] Centro de Estudos do Mar, Universidade Federal do Paraná, 83255-000, Pontal do Paraná, Brazil

[3] Sorbonne Université, CNRS, UMR7144, Team ECOMAP, Station Biologique de Roscoff, 29680, Roscoff, France.

[4] Sorbonne Université, CNRS, FR2424, Roscoff Culture Collection, Station Biologique de Roscoff, 29680, Roscoff, France.

[5] Département de Biologie, Institut de Biologie Intégrative et des Systèmes, Université Laval, Québec, QC G1V 0A6, Canada

[6] Department of Biosciences, University of Oslo, PO Box 1066 Blindern, 0316, Oslo, Norway.

[*]**Corresponding authors:**

Clarence Wei Hung Sim (clarencesim95@gmail.com)

Mailing address: Asian School of the Environment, Nanyang Technological University, 50 Nanyang Avenue, 639798, Singapore.

Adriana Lopes dos Santos (a.l.d.santos@ibv.uio.no)

Mailing address: Department of Biosciences, University of Oslo, PO Box 1066 Blindern, 0316, Oslo, Norway.

Short running title: Under-ice phytoplankton bloom

Date: April 30, 2025

Contents

1 **Supplementary Material and methods** 3

1.1 Study area . . . 3

1.2 Environmental and biological data . . . 3

1.3 DNA extraction, PCR amplification and sequencing . . . 3

1.4 Sequence processing, trophic mode allocation and culturability . . . 4

1.5 Data analysis and visualization . . . 5

2 **Supplementary Tables** 5

3 **Supplementary Figures** 9

1) Supplementary Material and methods

1.1 Study area

The Ice camp Green Edge field campaign was conducted on landfast sea ice located on the western coast of Baffin Bay. Baffin Bay is a seasonally ice-covered regional sea within the Canadian Arctic. As ice melts in spring, the sea ice edge retreats westwards from Greenland towards Canada. Water masses in Baffin Bay circulate counter-clockwise. Warm and salty Atlantic-derived waters enter the Bay through the Davis Strait, north of the Labrador Sea, and move northwards along the coast of Greenland (Tang et al., 2004). Cold Arctic-derived waters entering northern Baffin Bay move southwards along the coast of Canada, eventually flowing out of southwestern Baffin Bay (Münchow et al., 2015) (Figure 1).

1.2 Environmental and biological data

All ancillary physico-chemical and biological data obtained from the Green Edge project are available as raw data (Massicotte et al., 2019) and as formatted files (Massicotte et al., 2020). Photosynthetically active radiation (PAR) was computed from 19 discrete spectral irradiance wavelengths (380-875 nm) measured using an ICE-Pro (an ice flow version of the Compact-Optical Profiling System, C-OPS). Sea ice and under-ice water Chlorophyll *a* (Chl *a*) concentrations were obtained by high-performance liquid chromatography (HPLC). Water Chl *a* concentration (mg.m^{-2}) was depth-integrated from four discrete depths corresponding to water samples obtained in the first 60 m of the water column, while depth-integrated ice Chl *a* (mg.m^{-2}) was derived from the bottom 10 cm of the ice. Pico- and nano-phytoplankton cell abundance was measured using a BD Accuri^{TM} C6 flow cytometer as previously described in Massicotte et al. (2020).

1.3 DNA extraction, PCR amplification and sequencing

DNA was extracted using ZR Fungal/Bacterial DNA MiniPrep (Zymo Research, Irvine, CA, USA) following the manufacturer's instructions, and final DNA concentration was measured using PicoGreen™ (Thermo Fisher Scientific, Waltham, MA, USA). The 18S rRNA V4 hypervariable gene region (around 380 bp) was amplified with the primers TAReuk454FWD1 (forward, 5'-CCAGCASCYGCGGTAATTCC-3') (Stoeck et al., 2010) and V4 18S Next.Rev (reverse, 5'-ACTTTCGTTCTTGATYRATGA-3') (Piredda et al., 2017). Reaction mixtures (20 μL) were performed using 10 μL of Phusion High-

Fidelity PCR Master Mix® 2×, 0.3 μM final concentration of each primer, 3% DMSO, 2% BSA, 5 ng of template DNA and H_2O. Thermal conditions were as follows: 98°C for 5 min, followed by 25 cycles of 98°C for 20 s, 52°C for 30 s, 72°C for 90 s, and a final cycle of 72°C for 5 min. Samples were amplified in triplicate and pooled together. PCR purification, library preparation and amplicon sequencing was conducted at the GeT-PlaGe platform of GenoToul (INRAE Auzeville, France) using an Illumina Miseq and the 2 x 250 cycles Miseq kit version 2.

1.4 Sequence processing, trophic mode allocation and culturability

Sequences were processed with scripts written in the R language (R Core Team, 2020) using the *dada2* package (Callahan et al., 2016). Primer sequences were first removed with *cutadapt* version 2.8 (Martin, 2011) using the default parameters. Reads were filtered and trimmed using the filterAndTrim function with the following parameters: truncLen = c(230, 230), maxN = 0, maxEE = c(2, 2), and truncQ=10. Forward and reverse reads were merged with the mergePairs function and chimeric sequences removed with the removeBimeraDenovo function, both using default parameters. Amplicon Sequence Variants (ASVs) obtained with the dada2 function were labelled with the first 10 characters of the 40 character hash value of the sequence computed using the sha1 function from the R *digest* package (Eddelbuettel, 2021). ASVs were taxonomically assigned using assignTaxonomy function with PR^2 database version 5.0.1 (https://pr2-database.org, Guillou et al., 2012) as a reference. ASVs with low (< 80%) bootstrap support from the assignTaxonomy function at a given taxonomic level were reclassified to the next higher taxonomic level until the bootstrap value was ≥ 80%. ASVs assigned to non-protist taxa (e.g. metazoans) were removed. This included all taxa from divisions Metazoa, Fungi, Rhodophyta, classes Phaeophyceae, Embryophyceae, orders Bryopsidales, Ulotrichales, Dasycladales, Trentepohliales, Cladophorales and unidentified Opisthokonta. This resulted in a total of 2196 ASVs.

ASVs were then assigned to a trophic mode (photosynthetic, mixotrophic, heterotrophic, dinoflagellate) based on the database from Schneider et al. (2020). Only ASVs classified as photosynthetic and constitutive mixotrophic were further analysed in this study. Non-constitutive mixoplankton (i.e. those that do not have the innate ability to perform photosynthesis) were also not considered, resulting in the removal of Ciliophora and Rhizaria. Finally, dinoflagellates were not considered, since this group contains taxa that have a range of trophic modes, which can vary even within a given genus (Cohen et al., 2021). ASVs corresponding to taxa not present in the Schneider database were assigned to a trophic mode using a majority rule. For example, within the class Prymnesiophyceae, 15 out of the 23 listed taxa were assigned as mixotrophic and the other 8 as photosynthetic. All other

Prymnesiophyceae taxa not present in the Schneider database were then assigned as mixotrophic. The majority rule was only applied to groups that have at least 5 taxa in the Schneider database. A few higher level taxa that were not present in the Schneider data were assigned to a trophic mode based on the literature. A total of 428 ASVs were classified as photosynthetic and mixotrophic and further considered in this study. Finally, the number of reads in each sample was normalized by the median dataset sequencing depth (10,231 reads).

The similarity of ASVs to sequences of taxa available in cultures was determined by the –usearch_global option of *vsearch* with iddef = 2 against culture sequences from the PR^2 database version 5.0.1 (Guillou et al., 2012).

1.5 Data analysis and visualization

Data analysis was performed within R, using the following packages: *tidyr* (Wickham, 2021) and *dplyr* (Wickham et al., 2021) for filtering and organizing data; *ggplot2* (Wickham, 2016) for data visualization; *treemapify* (Wilkins, 2021) for treemaps, *ggridges* (Wilke, 2021) for density plots, *ggcharts* (Neitmann, 2021) for lollipop charts, and *patchwork* (Pedersen, 2020) for merging plots. The following color-vision-deficiency friendly palettes were used: BrBg, Blues, Reds and Set1 from *RColorBrewer* (Neuwirth, 2014) and Okabe-Ito from base R (R Core Team, 2020).

All codes and data used in this study can be found in https://github.com/clarencesimple/SIM_GreenEdge_IceCamp/tree/main.

2) Supplementary Tables

Table S1: Number of samples obtained from metaPR^2 (Dataset version 2.0) used for biogeographical distribution analysis

Region	No. of samples
Arctic	486
North temperate	1315
Tropical	196
South temperate	743
Antarctic	134

Table S2: Sympagic diatom taxa identified by metabarcoding and SEM (Figure S3). Genera and species with ≥ 80 bootstrap sequence support are listed. Percentage reads corresponds to the sum of DNA reads from taxon in ice over total photosynthetic DNA reads in ice. Cross marks (×) indicate identification by SEM.

Genus	species	n ASVs	reads(%)	SEM
Amphora	*Amphora* sp.	2	1.15	×
Attheya	*A. longicornis*	1	0.13	
	A. septentrionalis	2	3.32	
Bacillaria	*Bacillaria* sp.	13	3.62	
Chaetoceros	*C. cinctus*	1	0.02	
	C. contortus	1	0.001	
	C. decipiens	1	0.0006	
	C. neogracilis	1	0.31	
	Chaetoceros sp.	1	0.14	×
	Chaetoceros sp2	1	0.03	
Cylindrotheca	*C. closterium*	1	0.24	
	Cylindrotheca sp.	4	1.78	
Diploneis	*Diploneis* sp.	1	0.006	
Entomoneis	*Entomoneis* sp.	6	1.91	
	E. kjellmanii			×
Eucampia	*Eucampia* sp.	1	0.009	
Fallacia	*Fallacia forcipata*	1	0.01	×
Fragilaria	*Fragilaria* sp.	1	0.02	
Fragilariopsis	*F. cylindrus*	2	1.20	
Grammonema	*G. striatula*	1	0.007	
Haslea	*H. crucigera*	3	0.26	
Navicula	*Navicula* sp.	3	6.30	×
	N. trigonocephala			×
	N. cf. *directa*			×
	N. cf. *gelida*			×
	N. cf. *transitans* var.*derasa*			×
Nitzschia	*Nitzschia* sp.	5	2.26	×
Pauliella	*P. taeniata*	1	0.04	
Pinnularia	*P. quadratarea* var. *constricta*			×
Pleurosigma	*P. intermedium*	3	0.11	
	P. tuxbergii var. *rhomboides*			×
Porosira	*P. glacialis*	1	0.25	
	Porosira sp.	1	0.18	
Pseudo-nitzschia	*Pseudo-nitzschia* sp.	1	0.19	
Pseudogomphonema	*Pseudogomphonema* sp.	1	0.14	
Skeletonema	*Skeletonema* sp.	1	0.002	
Stauroneis	*Stauroneis* sp.	3	0.39	
Thalassiosira	*T. aestivalis*	1	0.05	
	T. antarctica	1	0.38	
	T. hispida	1	0.06	
	T. nordenskioeldii	1	1.70	
	Thalassiosira sp.	2	0.49	
	T. cf. *eccentrica*			×
	T. gravida			×
	T. pacifica			×

Table S3: Phytoplanktonic diatom taxa identified by metabarcoding and SEM (Figures S4 and S5). Genera and species with ≥ 80 bootstrap sequence support are listed. Percentage reads corresponds to the sum of DNA reads from taxa in water over total photosynthetic DNA reads in water. Cross marks (×) indicate identification by SEM.

Genus	species	n ASVs	reads(%)	SEM
Achnanthes	*Achnanthes sp.*			×
Amphora	*Amphora* sp.	2	0.30	×
Attheya	*A. longicornis*	1	0.005	
	A. septentrionalis	1	0.84	×
Bacillaria	*Bacillaria* sp.	10	0.73	
Chaetoceros	*C. cinctus*	1	0.05	
	C. contortus	1	0.05	
	C. debilis 1	1	0.01	
	C. decipiens	1	0.001	
	C. neogracilis	1	1.12	
	Chaetoceros sp.	7	2.55	
	Chaetoceros sp2	1	0.06	
Cocconeis	*Cocconeis* sp.			×
Cylindrotheca	*C. closterium*	1	0.03	
	Cylindrotheca sp.	3	0.62	
Detonula	*D. confervacea*	1	0.01	
Entomoneis	*Entomoneis* sp.	5	0.43	
	E. kjellmanii var. *kariana*			×
Eucampia	*Eucampia* sp.	1	0.005	
Fallacia	*F. forcipata*	1	0.005	
Fragilaria	*Fragilaria* sp.	4	3.84	
Fragilariopsis	*F. cylindrus*	2	6.03	
Grammonema	*G. striatula*	1	0.003	
Haslea	*H. crucigera*	2	0.11	
Licmophora	*L. gracilis*	1	0.001	
Mediolabrus	*Mediolabrus comicus*			×
Navicula	*Navicula* sp.	3	1.65	×
	N. superba			×
	N. cf. *pagophila* var *manitounukensis*			×
	N. trigonocephala var. *depressa*			×
Nitzschia	*Nitzschia* sp.	4	1.33	
Pauliella	*P. taeniata*	1	0.66	
Pinnularia	*Pinnularia quadratarea* var. *constricta*			×
Pleurosigma	*P. intermedium*	3	0.15	
	P. stuxbergii var. *rhomboides*			×
Porosira	*P. glacialis*	1	1.53	
	Porosira sp.	1	1.94	
Pseudo-nitzschia	*Pseudo-nitzschia* sp.	3	8.17	×
Pseudogomphonema	*Pseudogomphonema* sp.	1	0.11	
	P. arcticum			×
	P. septentrionale var. *angustatum*			×
Skeletonema	*S. marinoi*	1	0.004	
	Skeletonema sp.	1	0.13	
Stauroneis	*Stauroneis* sp.	2	0.17	
Thalassiosira	*T. aestivalis*	1	0.93	
	T. anguste-lineata	1	0.02	
	T. antarctica	1	2.93	
	T. gravida	1	0.43	×
	T. hispida	1	0.12	
	T. nordenskioeldii	1	1.93	×
	Thalassiosira sp.	3	2.92	×
	T. cf. *eccentrica*			×
	T. hyalina			×

Table S4: ANOSIM R and p values obtained by comparing samples clustered based on size fractions, substrate, and bloom stages

	R-value	p-value
**All samples**		
Size fraction (pico, nano, micro)	0.558	0.001
Substrate (ice:water)	0.460	0.001
**Ice samples**		
Size fraction (pico, nano, micro)	0.671	0.001
Bloom stages (I:II:III)	0.173	0.004
**Water Samples**		
Size fraction (pico, nano, micro)	0.726	0.001
Bloom stages (I:II:III)	0.273	0.001

3) Supplementary Figures

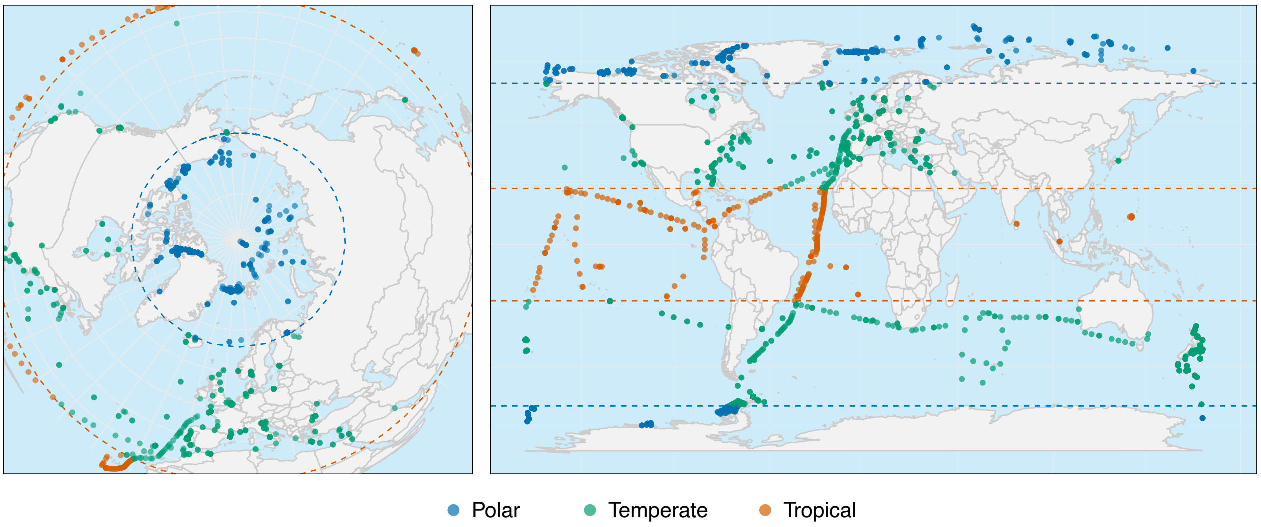

Figure S1: Pan-Arctic (left) and global (right) distribution of coastal and oceanic samples from metaPR^2 database (Datasets version: 2.0) used for biogeographical analyses in this study.

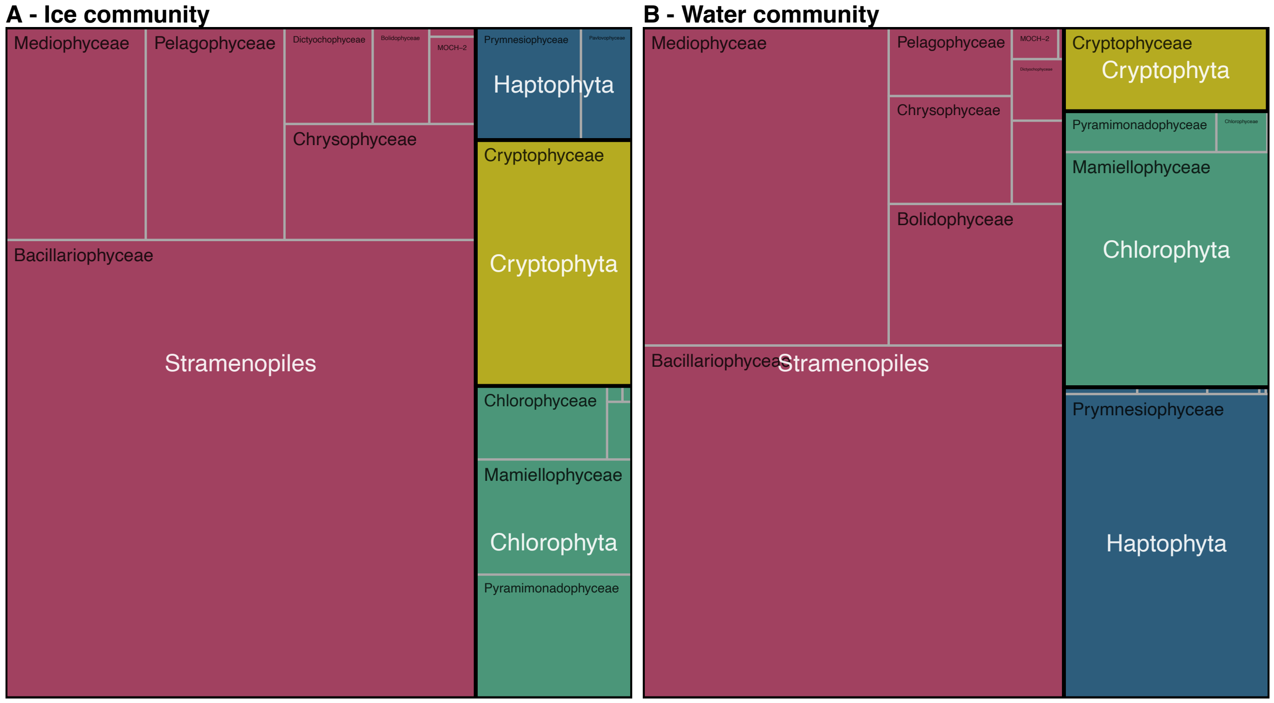

Figure S2: Community composition from most abundant photosynthetic taxa at class level from (A) ice and (B) water samples. Proportional area charts of normalised abundance of 18S V4 reads based on division (white labels) and class (black labels).

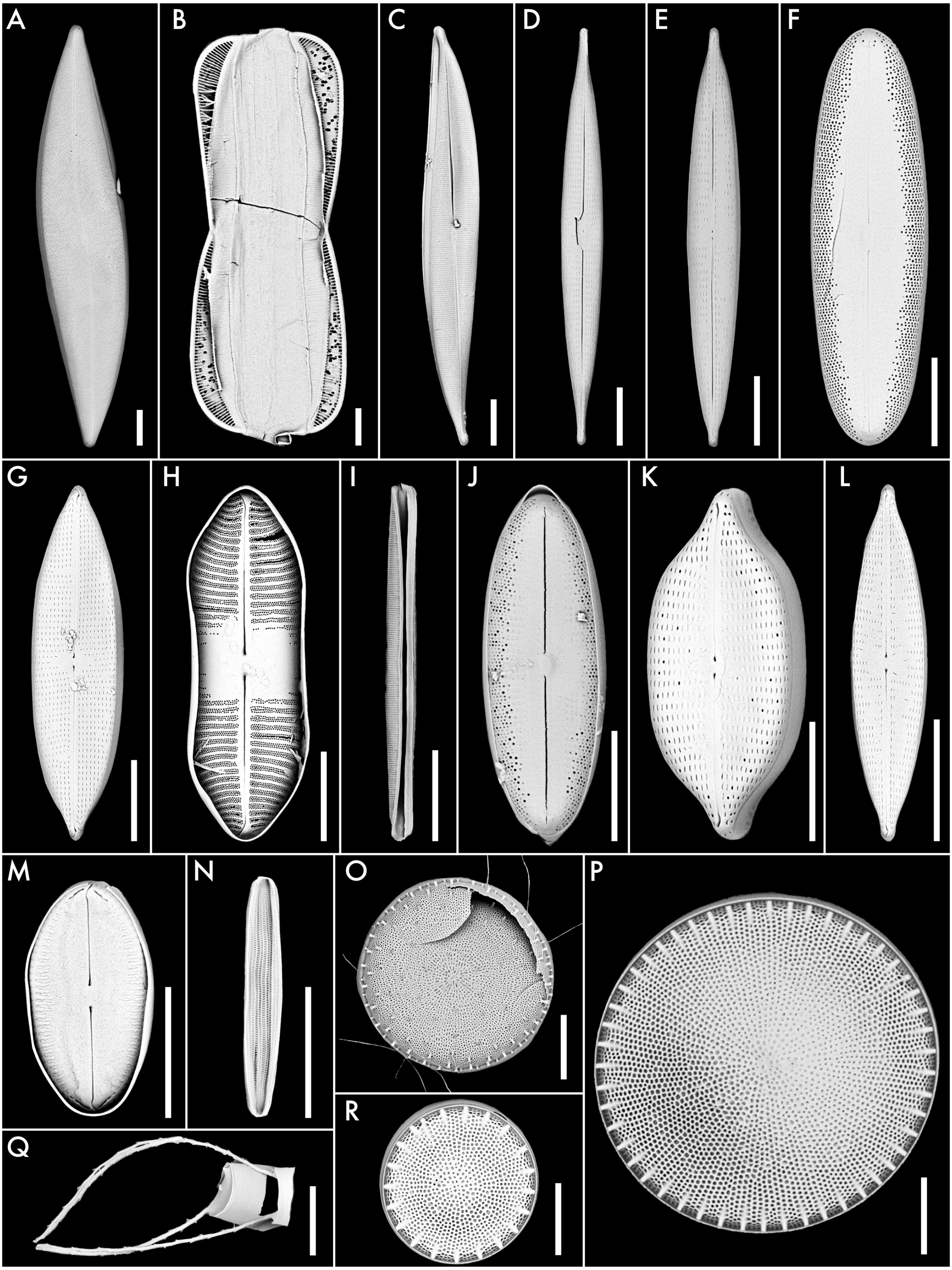

Figure S3: Diversity of diatoms in ice samples from scanning electron microscopy. Size bars correspond to 10 μm. A) *Pleurosigma stuxbergii* var. *rhomboides*; B) *Entomoneis kjellmanii*; C) *Gyrosigma concilians*; D) *Navicula* sp.; E) *Navicula* cf. *directa*; F) Raphid pennate G) *Navicula* cf. *gelida*; H) *Pinnularia quadratarea* var. *constricta*; I) *Nitzschia* sp.; J) cf. *Fallacia* sp.; K) *Navicula trigonocephala*; L) *Navicula transitans* var. *derasa*; M) *Fallacia* sp.; N) *Amphora* sp.; O) *Thalassiosira gravida*; P) *Thalassiosira pacifica*; Q) *Chaetoceros* sp.; R) *Thalassiosira* cf. *eccentrica.*

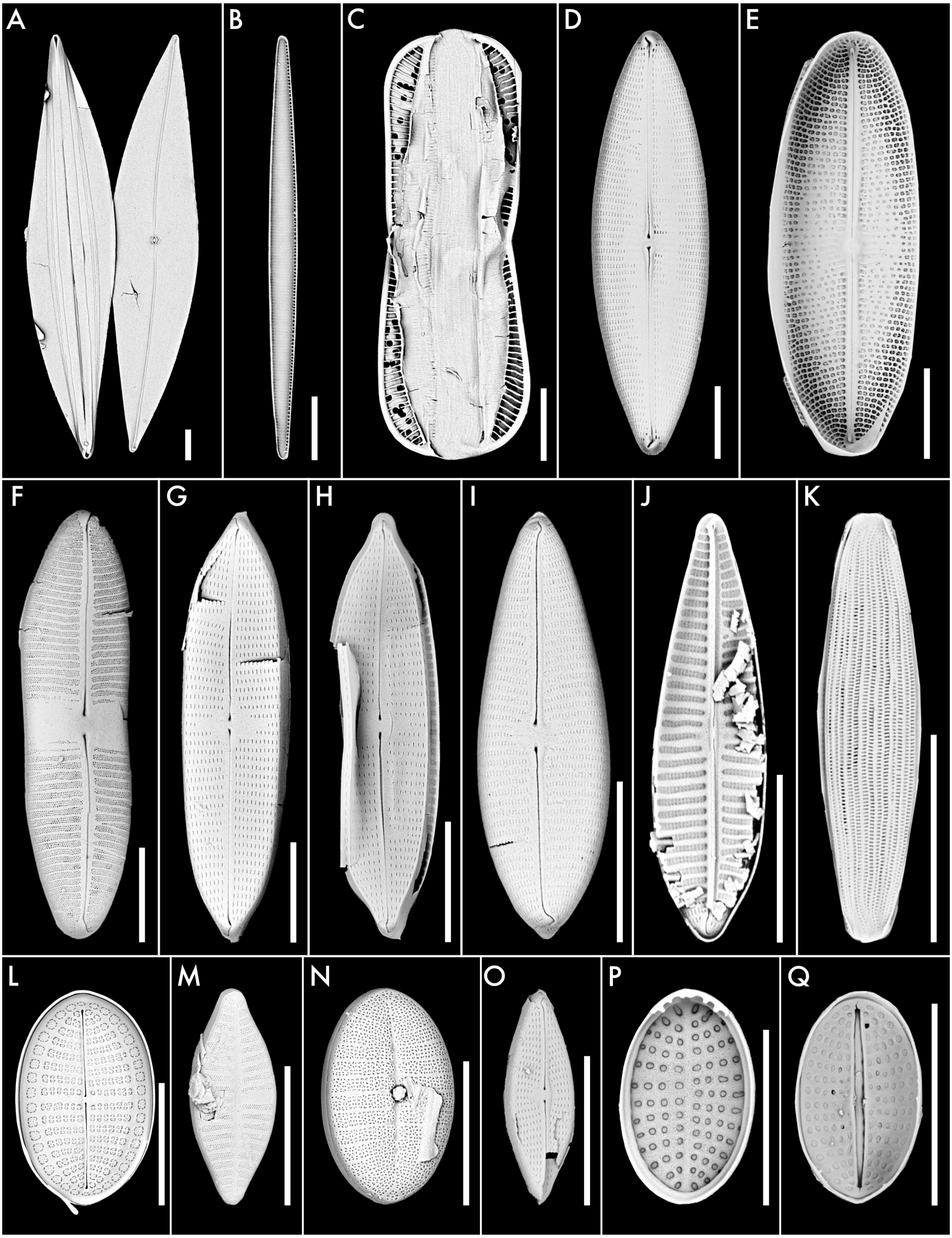

Figure S4: Diversity of pennate diatoms in water samples from scanning electron microscopy. Size bars correspond to 10 μm. A) *Pleurosigma stuxbergii* var. *rhomboides*; B) *Pseudo-nitzschia* sp.; C) *Entomoneis kjellmanii* var. *kariana*; D) *Navicula* sp.; E) *Navicula* cf. *pagophila* var. *manitounukensis*; F) *Pinnularia quadratarea* var. *constricta*; G) *Navicula trigonocephala* var. *depressa*; H) *Navicula trigonocephala* var. *depressa*; I) *Pseudogomphonemma arcticum*; J) *Pseudogomphonema septentrionale* var. *angustatum*; K) *Amphora* sp.; L) *Cocconeis* sp.; M) *Achnanthes* sp.; N) *Cocconeis* sp.; O) *Navicula* sp.; P) *Cocconeis* sp.; Q) *Cocconeis* sp.

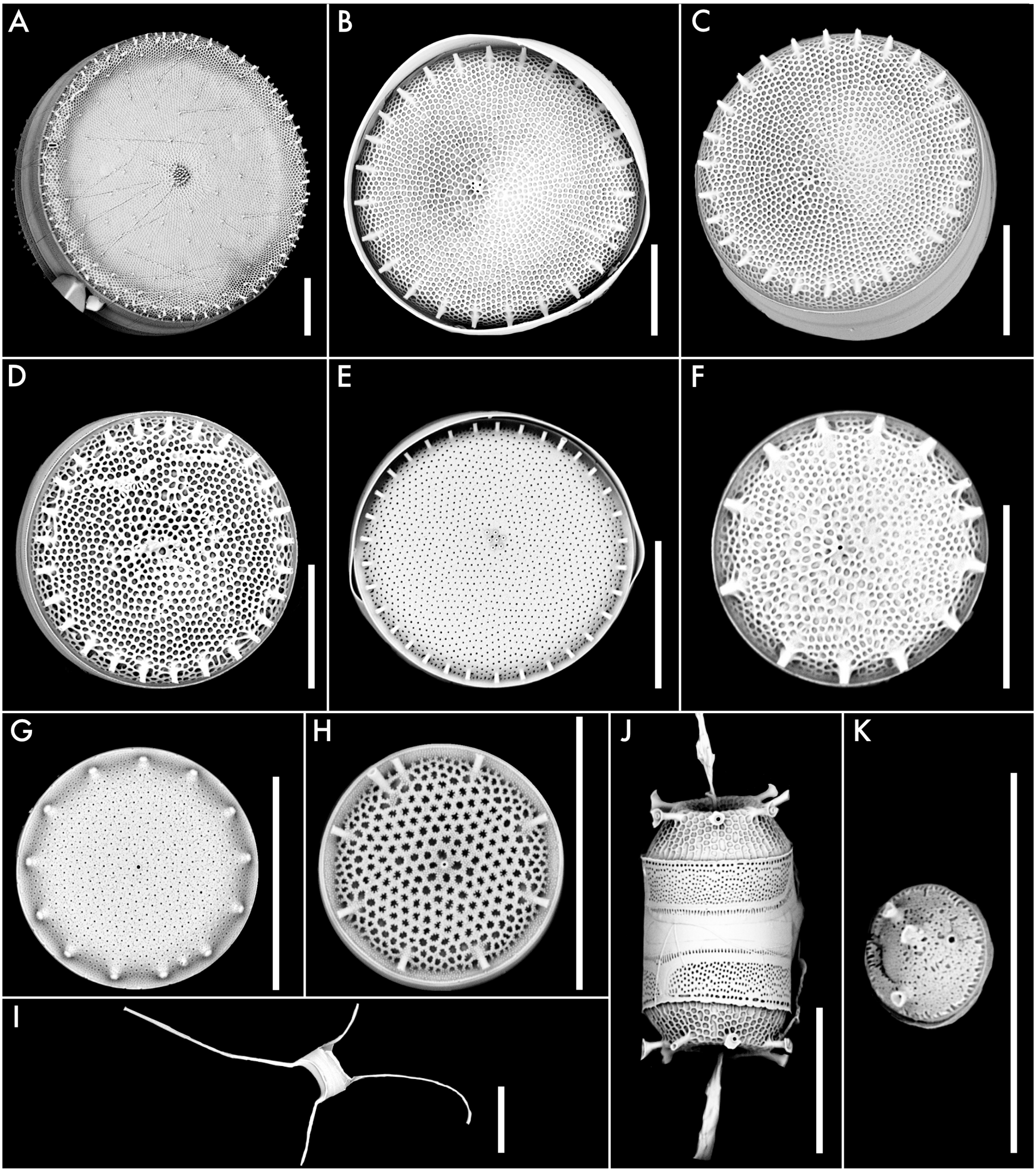

Figure S5: Diversity of centric diatoms in water samples from scanning electron microscopy. Size bars correspond to 10 μm. A) *Thalassiosira gravida*; B) *Thalassiosira* sp.; C) *Thalassiosira* cf. *eccentrica*; D) *Thalassiosira* sp.; E) *Thalassiosira hyalina*; F) *Thalsssiosira* sp.; G) *Thalassiosira* sp.; H) *Thalassiosira* sp.; I) *Attheya septentrionalis*; J) *Thalassiosira nordenskioeldii*; K) *Mediolabrus comicus*.

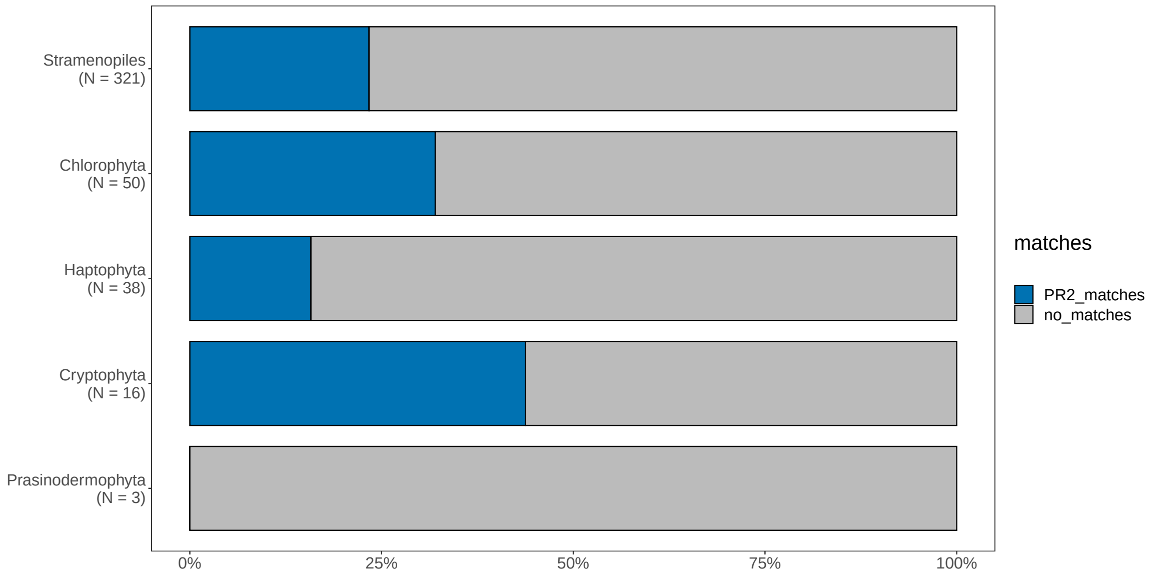

Figure S6: Proportion of ASVs within each division with 100% match to PR^2 sequences from cultures
.

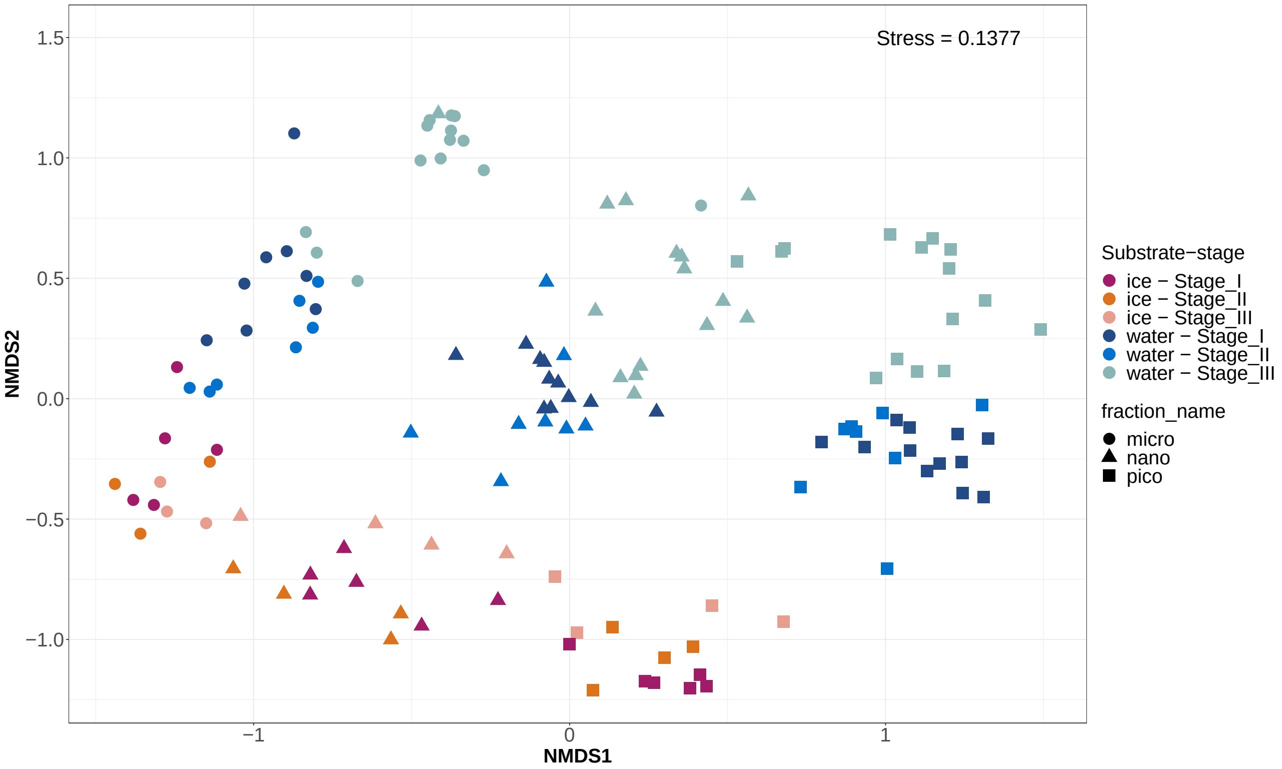

Figure S7: Non-metric multidimensional scaling (NMDS) analysis based on Bray-Curtis dissimilarities of the photosynthetic community composition at ASV level. Labels according to substrate-stage and size fraction.

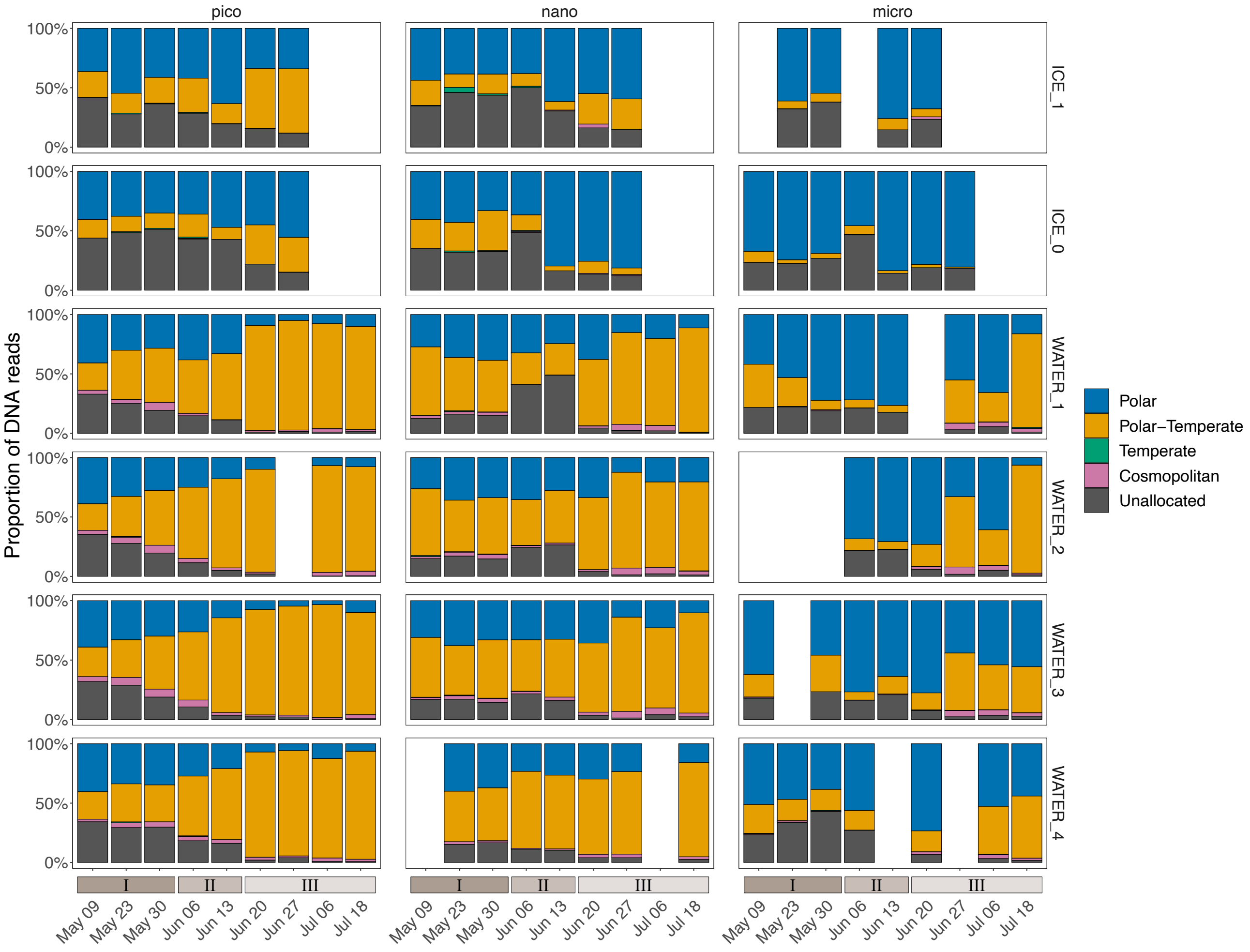

Figure S8: Temporal variation of ASVs according to their biogeographical distribution for each size fraction and each sampled layer.

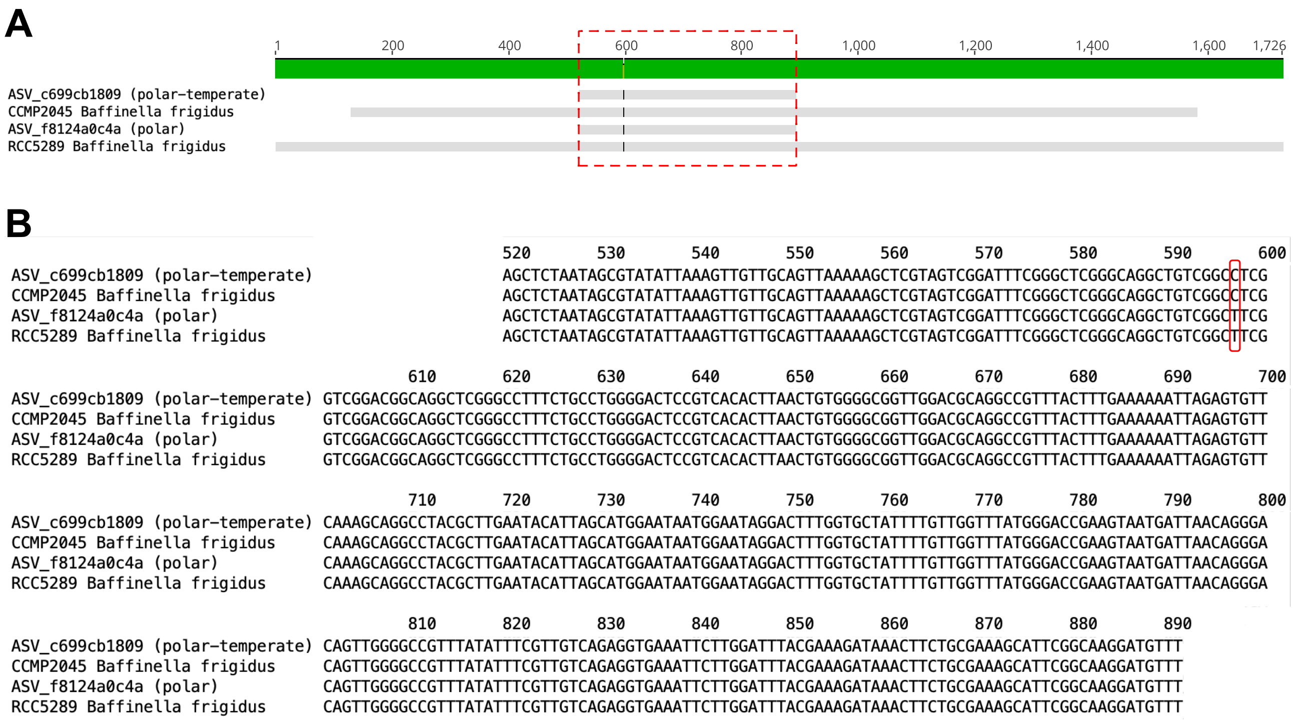

Figure S9: (A) DNA sequence alignment of two 18S V4 metabarcoding ASVs assigned to *Baffinella frigidus*, and the two partial 18S sequences from cultures. (B) Sequences from the red box showing single nucleotide differences at position 597. ASV_c699cb1809 has 100% similarity with CCMP2045 while ASV_f8124a0c4a has 100% similarity with RCC5289.

References cited

Callahan, B. J., McMurdie, P. J., Rosen, M. J., Han, A. W., Johnson, A. J. A., & Holmes, S. P. (2016). DADA2: High-resolution sample inference from Illumina amplicon data. *Nature Methods*, *13*, 581–583. https://doi.org/10.1038/nmeth.3869

Cohen, N. R., McIlvin, M. R., Moran, D. M., Held, N. A., Saunders, J. K., Hawco, N. J., Brosnahan, M., DiTullio, G. R., Lamborg, C., McCrow, J. P., Dupont, C. L., Allen, A. E., & Saito, M. A. (2021). Dinoflagellates alter their carbon and nutrient metabolic strategies across environmental gradients in the central Pacific Ocean. *Nature Microbiology*, *6*, 173–186. https://doi.org/10.1038/s41564-020-00814-7

Eddelbuettel, D. (2021). Digest: Create compact hash digests of R objects [R package version 0.6.29].

Guillou, L., Bachar, D., Audic, S., Bass, D., Berney, C., Bittner, L., Boutte, C., Burgaud, G., de Vargas, C., Decelle, J., del Campo, J., Dolan, J. R., Dunthorn, M., Edvardsen, B., Holzmann, M., Kooistra, W. H., Lara, E., Le Bescot, N., Logares, R., . . . Christen, R. (2012). The protist ribosomal reference database (PR^2): A catalog of unicellular eukaryote small sub-unit rRNA sequences with curated taxonomy. *Nucleic Acids Research*, *41*, D597–D604. https://doi.org/10.1093/nar/gks1160

Martin, M. (2011). Cutadapt removes adapter sequences from high-throughput sequencing reads. *EMBnet.journal*, *17*, 10–12. https://doi.org/10.14806/ej.17.1.200

Massicotte, P., Amiraux, R., Amyot, M.-P., Archambault, P., Ardyna, M., Arnaud, L., Artigue, L., Aubry, C., Ayotte, P., Bécu, G., Bélanger, S., Benner, R., Bittig, H. C., Bricaud, A., Brossier, É., Bruyant, F., Chauvaud, L., Christiansen-Stowe, D., Claustre, H., . . . Babin, M. (2019). Green edge ice camp campaigns: Understanding the processes controlling the under-ice arctic phytoplankton spring bloom. *SEANOE*. https://doi.org/10.17882/59892

Massicotte, P., Amiraux, R., Amyot, M.-P., Archambault, P., Ardyna, M., Arnaud, L., Artigue, L., Aubry, C., Ayotte, P., Bécu, G., Bélanger, S., Benner, R., Bittig, H. C., Bricaud, A., Brossier, É., Bruyant, F., Chauvaud, L., Christiansen-Stowe, D., Claustre, H., . . . Babin, M. (2020). Green Edge ice camp campaigns: Understanding the processes controlling the under-ice Arctic phytoplankton spring bloom. *Earth System Science Data*, *12*, 151–176. https://doi.org/10.5194/essd-12-151-2020

Münchow, A., Falkner, K. K., & Melling, H. (2015). Baffin Island and West Greenland Current Systems in northern Baffin Bay. *Progress in Oceanography*, *132*, 305–317. https://doi.org/10.1016/j.pocean.2014.04.001

Neitmann, T. (2021). *Ggcharts: Get you to your desired plot faster* [R package version 1.5.1.].

Neuwirth, E. (2014). *Rcolorbrewer: ColorBrewer palettes* [R package version 1.1-2].

Pedersen, T. L. (2020). *Patchwork: The composer of plots* [R package version 1.1.1].

Piredda, R., Tomasino, M. P., D'Erchia, A. M., Manzari, C., Pesole, G., Montresor, M., Kooistra, W. H., Sarno, D., & Zingone, A. (2017). Diversity and temporal patterns of planktonic protist assemblages at a Mediterranean Long Term Ecological Research site. *FEMS Microbiology Ecology*, *93*, 1–14. https://doi.org/10.1093/femsec/fiw200

R Core Team. (2020). *R: A language and environment for statistical computing*. R Foundation for Statistical Computing. Vienna, Austria.

Schneider, L., Anestis, K., Mansour, J., Anschütz, A., Gypens, N., Hansen, P., John, U., Klemm, K., Martin, J., Medic, N., Not, F., & Stolte, W. (2020). A dataset on trophic modes of aquatic protists. *Biodiversity Data Journal*, *8*, e56648. https://doi.org/10.3897/bdj.8.e56648

Stoeck, T., Bass, D., Nebel, M., Christen, R., Jones, M. D., Breiner, H. W., & Richards, T. A. (2010). Multiple marker parallel tag environmental DNA sequencing reveals a highly complex eukaryotic community in marine anoxic water. *Molecular Ecology*, *19*, 21–31. https://doi.org/10.1111/j.1365-294X.2009.04480.x

Tang, C. C., Ross, C. K., Yao, T., Petrie, B., DeTracey, B. M., & Dunlap, E. (2004). The circulation, water masses and sea-ice of Baffin Bay. *Progress in Oceanography*, *63*, 183–228. https://doi.org/10.1016/j.pocean.2004.09.005

Wickham, H. (2016). *Ggplot2: Elegant graphics for data analysis*.

Wickham, H. (2021). *Tidyr: Tidy messy data* [R package version 1.1.3].

Wickham, H., François, R., Henry, L., & Müller, K. (2021). *Dplyr: A grammar of data manipulation* [R package version 1.0.7].

Wilke, C. O. (2021). *Ggridges: Ridgeline plots in 'ggplot2'* [R package version 0.5.3].

Wilkins, D. (2021). *Treemapify: Draw treemaps in 'ggplot2'* [R package version 2.5.5].
